# Pharmacokinetics of Dibutyl Phthalate (DBP) in the Rat Determined by UPLC-MS/MS

**DOI:** 10.3390/ijms14010836

**Published:** 2013-01-04

**Authors:** Li-Wen Chang, Mei-Ling Hou, Tung-Hu Tsai

**Affiliations:** 1 Institute of Traditional Medicine, School of Medicine, National Yang-Ming University, Taipei 112, Taiwan; E-Mails: liwenchang104@gmail.com (L.-W.C.); maylinghou@gmail.com (M.-L.H.); 2 School of Pharmacy, College of Pharmacy, Kaohsiung Medical University, Kaohsiung 807, Taiwan; 3 Department of Education and Research, Taipei City Hospital, Taipei 106, Taiwan

**Keywords:** dibutyl phthalate (DBP), liquid chromatography with tandem mass spectrometry (LC-MS/MS), pharmacokinetics, plasticizer

## Abstract

Dibutyl phthalate (DBP) is commonly used to increase the flexibility of plastics in industrial products. However, several plasticizers have been illegally used as clouding agents to increase dispersion of aqueous matrix in beverages. This study thus develops a rapid and validated analytical method by ultra-performance liquid chromatography with tandem mass spectrometry (UPLC-MS/MS) for the evaluation of pharmacokinetics of DBP in free moving rats. The UPLC-MS/MS system equipped with positive electrospray ionization (ESI) source in multiple reaction monitoring (MRM) mode was used to monitor *m*/*z* 279.25→148.93 transitions for DBP. The limit of quantification for DBP in rat plasma and feces was 0.05 μg/mL and 0.125 μg/g, respectively. The pharmacokinetic results demonstrate that DBP appeared to have a two-compartment model in the rats; the area under concentration *versus* time (AUC) was 57.8 ± 5.93 min μg/mL and the distribution and elimination half-life (t_1/2,α_ and t_1/2,β_) were 5.77 ± 1.14 and 217 ± 131 min, respectively, after DBP administration (30 mg/kg, i.v.). About 0.18% of the administered dose was recovered from the feces within 48 h. The pharmacokinetic behavior demonstrated that DBP was quickly degraded within 2 h, suggesting a rapid metabolism low fecal cumulative excretion in the rat.

## 1. Introduction

**Figure 1:**
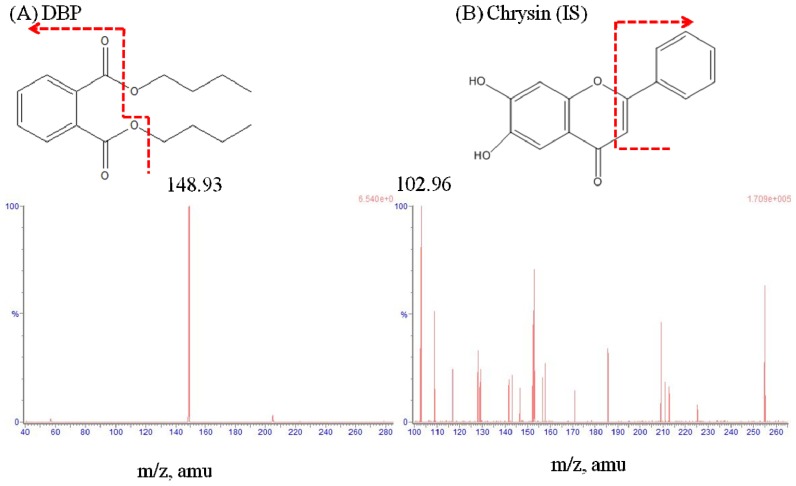
Representative product ion mass spectra and chemical structures of (**A**) Dibutyl phthalate (DBP) and (**B**) chrysin (internal standard).

Phthalate ester derivatives have been used as plasticizers in a wide variety of plastic products, such as children’s toys, medical devices and various types of packaging [1]. However, in some illegal food industry applications, phthalates have been used as a low-cost substitute to replace palm oil as a clouding agent added to juices, yogurt and beverages. Dibutyl phthalate (DBP; Figure 1) is one of the most popular phthalates used as clouding agent in beverages, which has been detected by the Taiwan Food and Drug Administration in 2011 [2].

Previous research has demonstrated that DBP is a developmental and reproductive toxicant that can reduce the binding of estrogen to estrogen receptors and inhibit estrogen’s transcriptional activity [3]. Toxicity studies have investigated the developmental toxicity of DBP in rodents. Dobrzynska *et al.*, (2011) indicated that paternal DBP exposure may disturb offspring, including delay of female sexual maturation, deterioration of the quality of sperm [4] and malformation of the anorectum in males [5]. In addition, oral DBP administration appears to affect the second generation more than the first generation in rats [6]. Concerning potential testicular toxicity of DBP in rodents, previous reports have indicated that exposure to DBP results in significantly lower testicular weight [7], reduced anogenital distance [8], spermatogenesis dysfunction [9] and cryptorchidism [10] in male rats.

Although considerable research attention has been given to male reproductive toxicity of DBP in rodents, most other studies focus on the pharmacokinetics of DBP in pregnant rats and their offspring [11–13]. Recently, phthalates have been found to be prevalent in various environments, including food, air, soil and even in pharmaceutical products and medical devices [14]. From all these sources, the chemical is taken up into humans and animals primarily via chronic absorption by ingestion, inhalation or dermal absorption and by acute intake via injection of pharmaceutical products or medical devices. Therefore, the pharmacokinetics of plasticizer absorbed by acute and chronic methods must be assessed.

To measure the phthalates, several quantitative methods have been developed, including high-performance liquid chromatography with ultraviolet detection (HPLC-UV) [15], gas chromatography with flame ionization detection (GC-FID) [16], gas chromatography employing an electron capture detector (GC-ECD) [17] and gas chromatography-mass spectrometry (GC-MS) [18]. High-performance liquid chromatography with tandem mass spectrometry (HPLC-MS/MS) has been used for the quantitative analysis of ^13^C_4_-labeled phthalates and their metabolites in bio-samples [1,19]. However, this method may be time-consuming and complicated in the pre-treatment for analysis of ^13^C_4_-labeled phthalates and their metabolites. Nowadays, LC-MS/MS is the main trend for bioanalysis. To compromise the advantages of the isotope-labeled analysis, the detailed and completed method validation should be performed according to US FDA guidelines [20].

The novelty of this article is to develop a freely moving rat model coupled to LC-MS/MS to investigate the pharmacokinetics of DBP with oral and intravenous administration in rats. To investigate the oral absorption and fecal excretion of DBP, the aim of this study is to optimize a sensitive ultra-performance liquid chromatography coupled to tandem mass spectrometry (UPLC-MS/MS) to measure DBP in rat plasma and feces in free moving male rats. This method is then applied to the pharmacokinetics of DBP and its unabsorbed fecal elimination.

## 2. Results and Discussion

### 2.1. LC–MS/MS and Method Validation

In order to improve the detection of DBP, the UPLC–MS/MS condition has been optimized and validated by the following steps. First, an intellstarte (Waters Acquity UPLC™ system built-in software) was used to optimize the mass condition for DBP and the internal standard. Two types of mobile phase, 0.1% formic acid-methanol and ammonium acetate (5 mM)-methanol, were used to adjust the analytes’ signal strength to an acceptable mass spectrometer condition. Secondly, since the mass condition was performed in positive ionization mode, the aqueous phase with ammonium acetate (5 mM) was selected. For the organic phase, acetonitrile and methanol have both been tested. A gradient elution consisted with a mobile phase from ammonium acetate (5 mM)-methanol (10:90, *v*/*v*) to ammonium acetate (5 mM)-methanol (30:70, *v*/*v*) was adjusted at a flow-rate of 0.25 mL/min. Based on the results from the above tests, the optimized mobile phase decided for the optimal LC condition consisted of ammonium acetate (5 mM)-methanol (27:73, *v*/*v*) at a flow-rate of 0.25 mL/min. Thirdly, several methods for sample preparation, such as protein precipitation and liquid–liquid extraction, have been examined. However, background interference was presented in the blank plasma extract by liquid-liquid extraction, but did not appear in the process of protein precipitation.

In the universal background presence of various plasticizers in the general environment, however, the contamination level can be reduced by careful selection of analytical tools, glassware, solvents and frequent verification of the chromatographic system [21]. The process of liquid-liquid extraction was more complicated than protein precipitation, and we also found that the blank plasma extract was present from the background interference by liquid-liquid extraction. Therefore, we simplified the sample preparation procedure by using protein precipitation with methanol to avoid any possibility of DBP contamination. The potential hydrolyze enzymes were also removed by protein precipitation. The *ex vivo* study of the hydrolytic activity was dependent on the protein concentration in the supernatant, optimum temperature and pH of the hydrolysis, which was 50 °C and 8.2, respectively [22], and the enzymatic kinetics were allowed to establish a reaction time of 30 min [23]. Also, the protein precipitation didn’t take a long time, and the mixture was centrifuged and kept at 4 °C.

**Figure 2:**
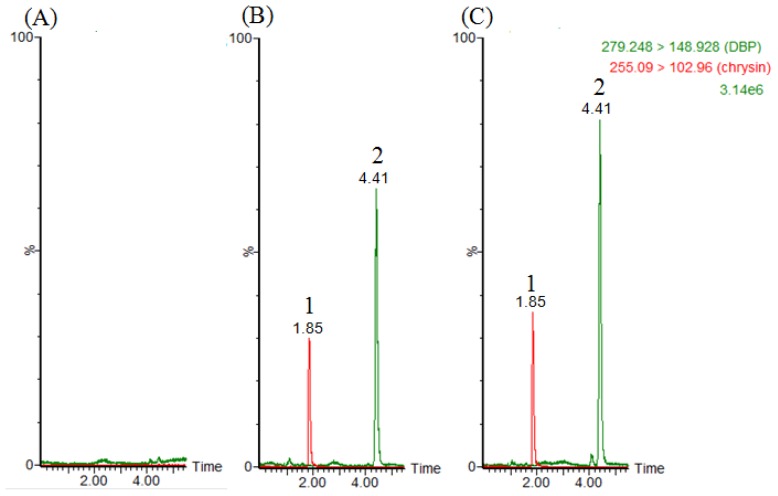
Chromatograms of (**A**) blank plasma after protein precipatation, (**B**) blank plasma spike with DBP (1 μg/mL) and chrysin (1 μg/mL) and (**C**) real sample collected at 5 min after DBP administration (30 mg/kg, i.v.) with chrysin (1 μg/mL). 1: chrysin (internal standard), 2: DBP.

**Figure 3:**
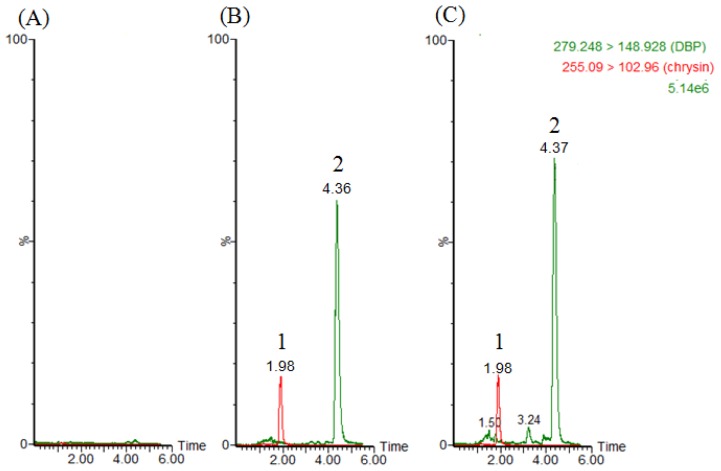
Chromatograms of (**A**) blank feces after protein precipatation (**B**) blank feces spike with DBP (1 μg/g) and chrysin (1 μg/mL) and (**C**) real sample collect at 0–12 h after DBP administration (100 mg/kg, p.o.) with chrysin (1 μg/mL). 1: chrysin (internal standard), 2: DBP.

Figures 2A and 3A show chromatograms of blank samples (plasma, feces) after protein precipatation, illustrating the baseline without any disturbing peaks in the chromatograms of real samples. No interference or other metabolites were co-eluted with DBP. Figure 2B shows a chromatogram of a plasma sample spike with DBP (1 μg/mL) and chrysin (1 μg/mL), and Figure 2C shows the real blood samples after DBP administration (30 mg/kg, i.v.) with chrysin (1 μg/mL).

For fecal analysis, Figure 3B shows the chromatogram of fecal sample spiked with DBP (1 μg/g) and chrysin (1 μg/mL), and Figure 3C shows the actual fecal sample collected from rat feces (0–12 h) after DBP administration (100 mg/kg, p.o.) with chrysin (1 μg/mL).

To assess the analytical range of the developed method, various concentrations of DBP in blank samples (plasma, feces) ranging from 0.05 to 2.5 μg/mL and 0.125 to 2.5 μg/g were tested. The results showed a good correlation coefficient (*r*^2^ ≥ 0.995) for DBP over the concentration range from separately prepared analytical runs on different days. As shown in Tables 1 and 2, five replications of each freshly prepared calibration standards were used for the determination of intra-day and inter-day accuracy (Bias%) and precision (R.S.D.%). The inter- and intra-day precision and accuracy values for blood and feces are presented in Tables 1 and 2, respectively. For DBP in rat plasma, the inter-day precisions (R.S.D.%) ranged from 0.02% to 15.97%, while accuracy (Bias%) was between (–7.27%) and 6.07%. Intra-day precision (R.S.D.%) ranged from 0.07% to 15.27%, and accuracy (Bias%) was between (−7.16%) and 10.15%. Likewise, the inter-day precisions (R.S.D.%) ranged from 1.05% to 11.86%, and accuracy (Bias%) were (−3.71%) to 11.93% for DBP in rat feces. On the other hand, intra-day precision (R.S.D.%) ranged from 0.82% to 17.50%, and accuracy (Bias%) was (−6.43%) to 14.23%. The inter- and intra-day accuracy and precision showed repeatability and reliability. The calibration curves of DBP in plasma and feces samples were acceptable for quantification, and the analytical method could be applied to pharmacokinetic studies. The data demonstrated that the LOQ of DBP in plasma samples and fecal samples was 0.05 μg/mL and 0.125 μg/g, respectively.

Analyte matrix effect and recovery were used to evaluate different sample preparations, while the post-column infusion method and the post-extraction spike method were used to evaluate matrix effects. In this study, the post-extraction spike method was selected to evaluate the matrix effect of DBP in biosamples (plasma, feces). This method quantitatively evaluates matrix effects by contrasting the response of an analyte in standard solution to the response of the analyte spiked into a blank matrix sample that has been passed through the sample preparation process [24].

The data demonstrated that the matrix effect of DBP in rat plasma and feces were 98.50 ± 0.54 and 100.63% ± 0.08%, respectively (Table 3). The extraction recovery of DBP in rat plasma and feces were 99.02 ± 2.22 and 99.4% ± 0.77%, respectively (Table 3). In addition, chrysin had a high matrix effect and recovery in rat plasma and feces after protein precipitation, though it was observed that chrysin did not interfere with the matrix effect and recovery of DBP in rat plasma and feces (Table 3). As a result, it was accepted as the internal standard for this study. The results showed that this analytical method was acceptable for use in the pharmacokinetic study of DBP in freely moving adult male rats.

### 2.2. Pharmacokinetics Study of DBP

**Figure 4:**
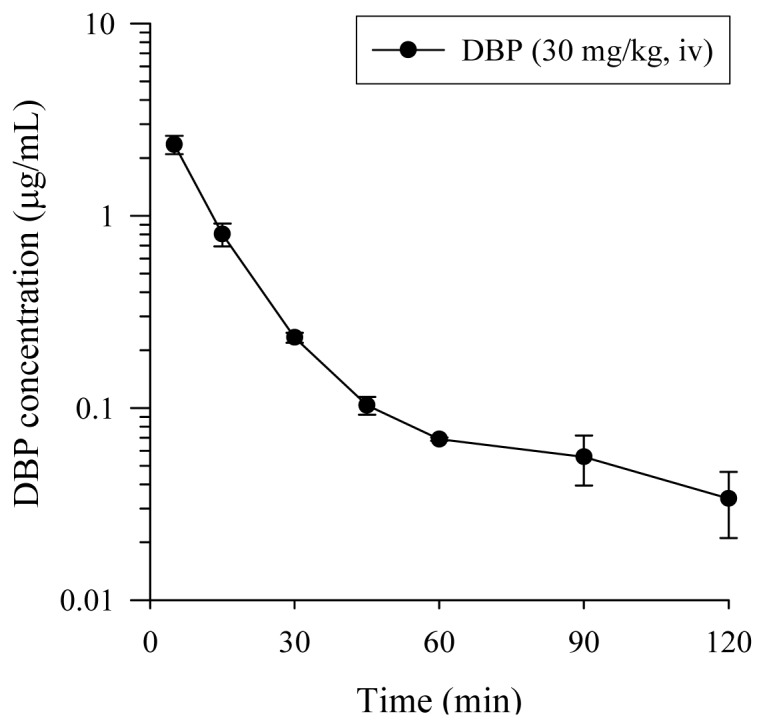
Concentration *versus* time curve in rat plasma after DBP (30 mg/kg, i.v.) administration. Data are presented as mean ± SEM (*n* = 5).

The pharmacokinetic models (one- *versus* two-compartment) were compared according to the AIC [25], with minimum AIC values being regarded as the best representation of the blood concentration-time course data. A compartmental model with individual animal data after dose was proposed by the computer program WinNonlin. This AIC value on average decreases from (−23.57) ± 3.66 for one-compartment model to (−37.09) for the two-compartment model, indicating that the two-compartment model is more suitable than the one-compartment model for the DBP administration (30 mg/kg, i.v.; Figure 4; Table 4). The following equation applies to a two-compartmental pharmacokinetic model: *C* = *A*e^−α^*^t^* + *B*e^−β^*^t^*. In the equation, *A* and *B* are the concentration (*C*) intercepts for fast and slow disposition phases, respectively; α and β are the disposition rate constants for fast and disposition phases, respectively. Analysis of the data after DBP administration (30 mg/kg, i.v.) yields the pharmacokinetic equation of *C* = 6.04e^−0.15^*^t^* + 0.18e^−0.02^*^t^*.

**Figure 5:**
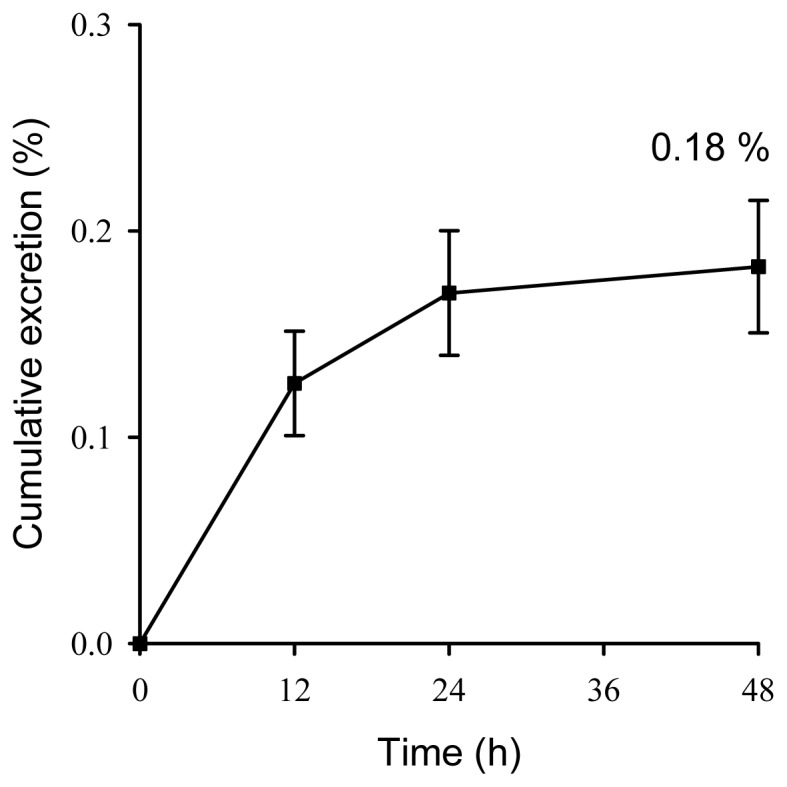
Cumulative excretion of DBP in feces after oral administration of DBP (100 mg/kg). Data are expressed as mean ± SEM (*n* = 6).

The present study first uses a freely moving rat model to investigate the pharmacokinetics of DBP with oral and intravenous administration to rats. The pharmacokinetic curve showed that the mean plasma concentration was rapidly distributed during the first 60 min and then trends toward slow excretion after DBP administration (30 mg/kg, i.v.; Figure 4). The pharmacokinetic data indicated that DBP achieved an AUC of 57.8 ± 5.93 min μg/mL, a clearance of 551 ± 64 mL/min/kg and a mean residence time of 123 ± 78 min. The distribution and elimination half-life were 5.77 ± 1.14 and 217 ± 131 min, respectively, showing DBP rapid distribution and slow elimination after DBP administration (30 mg/kg, i.v.; Table 4). However, we could not detect DBP in plasma up to six hours after a single oral dose was administered (100 mg/kg, p.o.), indicating that DBP was metabolized rapidly through the gastrointestinal tract in rats, and only 0.18% of the administered dose was recovered from the feces within 48 h (Figure 5). The hypothesis for the disposition of phthalates in the body may proceed through the following three major steps. In the first step, the phthalate diesters will be cleaved into the individual hydrolytic monoesters. In the second step, the alkyl chain of the resulting hydrolytic monoester can be modified by various oxidation reactions. In the final step, both the hydrolytic monoester and the oxidized secondary metabolites can be conjugated with glucuronic acid and excreted in urine [2,14]. Our result is consistent with the above hypothesis that DBP was rapidly distributed with a distribution half-life of 5.77 ± 1.14 min after DBP administration (30 mg/kg, i.v.) and undetectable in the plasma and low cumulative fecal excretion after oral administration (100 mg/kg, p.o.).

Due to the illegal use of DBP (one of the plasticizers) in the food industry, detailed toxicokinetic information may not be sufficient. We have surveyed from the previous literature focusing on the dose selection of oral and IV administration [12,26]. However, the correlation between drug concentrations and enzymes were still insufficient. The DBP exposure levels of the pharmacokinetic parameters, with the rate limited by the enzyme detoxification systems, were limited.

For the low level of cumulative fecal excretion, the potential explanation is that the DBP may well be absorbed and quickly metabolized. Our results agree with a previous report that the fecal excretion is low and that more than 90% of the dose is excreted via metabolites in the urine within 48 h following either intravenous or oral administration [27]. For metabolism and elimination of DBP, previous studies have found that DBP is metabolized to its metabolite-monobutyl phthalate (MBP) through phase I biotransformation in the stomach and small intestine, and almost all enters the bloodstream as MBP [28]. Then, the phase II biotransformation of MBP is rapidly conjugated to its glucuronide conjugate (monobutyl phthalate glucuronide, MBP-G) within 5 min in maternal plasma [26].

## 3. Experimental Section

### 3.1. Chemicals

DBP was purchased from Accu Standard (Unitech, New Haven, CT, USA). Sodium chloride (NaCl), heparin sodium and olive oil were obtained from Sigma-Aldrich (St. Louis, MO, USA). Polyethylene glycol 400 (PEG 400) was purchased from Fluka (St. Louis, MO, USA). Water for all preparations was prepared by the Milli-Q system (Millipore, Milford, MA, USA). Ammonium acetate and methanol of MS grade were purchased from E. Merck (Darmstadt, Germany).

### 3.2. Animal Experiment

Adult male Sprague-Dawley rats weighing 200 ± 20 g were obtained from the National Yang-Ming University Animal Center, Taipei, Taiwan. The rats were specifically pathogen-free and had free access to food (Laboratory Rodent Diet 5001, PMI Nutrition International LLC, St. Louis, MO, USA) and water. The rats were housed with a 12-h light and 12-h dark cycle. All experimental protocols involving animals were reviewed and approved by the Institutional Animal Care and Use Committee (IACUC number: 1000902) of National Yang-Ming University. The rats were anesthetized by pentobarbital (50 mg/kg, i.p.), and polyethylene tubes were implanted in the right jugular and right femoral veins. The catheter was exteriorized, fixed in the dorsal neck region and capped with a stopper. For the oral administration group, only the right jugular was catheterized for blood sampling. The patency of the tubing was maintained by flushing with heparinized normal saline (0.9% NaCl, *w*/*v*, solution containing 20 IU/mL heparin sodium salt). While remaining anesthetized during a series of surgeries, each rat had its body temperature maintained at 37 °C with a heating pad. After surgery, the rat was placed in an experimental cage and allowed to recover for one day. During the recovery period, the rat was kept warm under a light.

### 3.3. LC–MS/MS and Method Validation

A Waters Acquity UPLC™ system coupled to a WatersXevo™ tandem quadrupole mass spectrometer fitted with electrospray ionization probe (Waters Corporation, Milford, MA, USA) was used in this study. The analytes were separated by an Acquity UPLC BEH C18 (2.1 mm × 100 mm, 1.7 μm) column (Waters Corporation), and the column temperature was maintained at 40 °C. The mobile phase was optimized as ammonium acetate (5 mM)-methanol (27:73, *v*/*v*) at a flow-rate of 0.25 mL/min. The positive ion mode with multiple reactions monitor (MRM) was used for UPLC-MS/MS analysis. The following precursor to product ion transitions were used: *m*/*z* 279.25→148.93 for DBP and *m*/*z* 255.09→102.96 for the chrysin (internal standard, IS) (Figure 1). The following parameters were optimized for DBP and chrysin analysis: capillary voltage of 3.11 kV, desolvation gas (nitrogen) heated to 400 °C and a desolvation gas flow rate of 800 L/h. The cone voltages were set to 22 V and 66 V, while the collision energy voltages were set to 14 eV and 32 eV for DBP and chrysin, respectively (Table 5). The MassLynx 4.1 software (Waters Corporation, Milford, MA, USA) was used for data processing.

Pharmacokinetic studies for bioanalytical assays were based on the method validation according to the FDA guidelines [20], including tests of calibration curve, precision and accuracy. The limit of quantification (LOQ) was defined as the lowest concentration of the linear range, and the limit of detection (LOD) was defined as the concentration of analyte giving a signal-to-noise ratio (S/N) of 3. Quantitation of DBP in plasma and fecal fluid was based on calibration curves, ranging from 0.05 to 2.5 μg/mL and 0.125 to 2.5 μg/g with the correlation coefficient (*r*^2^) greater than 0.995, respectively.

The matrix effect and recovery of DBP in rat plasma and fecal fluid were assessed at the concentrations of 0.125, 1.25 and 2.5 μg/mL (μg/g). The chrysin (IS) concentrations were 1 μg/mL in rat plasma and fecal fluid. Matrix effect (%) and recovery (%) were calculated using the formulas:

(1) $$\begin{array}{c} \text{Matrix effect}=(\text{the peak areas for standards spiked}\;\text{after extraction}/\text{the peak areas}\\ \text{obtained in neat solution standards})\times 100 \end{array}$$

(2) $$\begin{array}{c} \text{Recovery}=(\text{the peak areas for standards spiked before extraction}/\text{the peak areas for}\\ \text{standards spiked after extraction})\times 100 \end{array}$$

### 3.4. Blood Sampling Collection

DBP was dissolved in PEG 400 and olive oil for intravenous and oral administration, respectively. According to a study done by Brunner *et al.*, dietary oils (corn oil, olive oil, sesame oil and soybean oil) intake may affect hepatic cytochrome P450 isoforms activity in rat [29]. However, there are no direct toxicity data between the corn oil and olive oil. Therefore, the olive oil was used as a vehicle for DBP oral administration [30]. For the intravenous group, DBP (30 mg/kg, *n* = 5) was given via the femoral vein catheter, whereas DBP (100 mg/kg, *n* = 6) was given via gastric gavage for the oral groups. An aliquot of 150 μL blood collected from the jugular vein catheter was withdrawn into a heparin-rinsed vial, and the lost blood was replaced with an equal volume (150 μL) of heparinized saline after each sampling. In the group with intravenous administration, blood samples were collected at 5, 15, 30 and 45 min and 1, 1.5 and 2 h after dosing. After the oral dose of 100 mg/kg, blood samples were collected at 15, 30 and 45 min and 1, 1.5, 2, 4 and 6 h after administration. Each blood sample was centrifuged at 6000× *g* for 10 min to acquire the plasma. The resulting plasma sample was stored at −20 °C before analysis.

### 3.5. Fecal Excretion of DBP after a Single Oral Administration

The fecal samples were collected separately at 12 h intervals for two consecutive days by a metabolic cage (Mini Mitter, Bend, OR, USA) after oral administration of DBP (100 mg/kg, *n* = 6). During this experiment, the rats also had free access to food and water in the animal facilities. The fecal sample was extracted with acetonitrile (1 g:4 mL, *w*/*v*) by homogenization for 1 min.

### 3.6. Blood and Fecal Sample Preparation

The protein precipitation method was used to remove macromolecules from plasma and fecal samples. An aliquot of 45 μL plasma and fecal sample or blank plasma and feces, 5 μL chrysin (IS) and 150 μL methanol were added and vortexed for 30 s. The mixture was centrifuged at 16,000× *g* for 10 min at 4 °C. Then, the supernatant was transferred to autosampler vials and 5 μL was injected into the UPLC system.

### 3.7. Pharmacokinetic Data Analysis and Statistics

All pharmacokinetic analysis was processed by the WinNonlin Standard Edition Version 1.0 (Scientific Consulting Inc.: Apex, NC, USA). Akaike Information Criterion (AIC) value was used to select the compartment model used for data analysis.

## 4. Conclusions

This study developed a sensitive and reliable analytical method to detect DBP in a freely moving rat model using a simple protein preparation detected by UPLC-MS/MS. The pharmacokinetic results revealed that DBP was best fitted by the two-compartment model in the rats. DBP was eliminated rapidly after intravenous and oral administration. The feces were a minor route of excretion of DBP in rats. These results provide very important information on the pharmacokinetics for further risk assessment for DBP.

## Figures and Tables

**Table 1:** Method validation for the intra-assay and inter-assay precision (R.S.D. %) and accuracy (Bias %) of the UPLC-MS/MS method for the determination of DBP in rat plasma.

Nominal concentration (μg/mL)	Precision (R.S.D.%)	Accuracy (Bias%)
**Inter-day**		

0.05	15.97	−1.17
0.1	11.87	−7.27
0.25	8.23	−2.57
0.5	4.70	6.07
1	1.33	−1.67
2.5	0.02	0.02

**Intra-day**		

0.05	15.27	10.15
0.1	6.36	−7.16
0.25	4.58	−0.79
0.5	8.10	5.10
1	2.59	−1.69
2.5	0.07	0.04

**Table 2:** Method validation for the intra-assay and inter-assay precision (R.S.D.%) and accuracy (Bias%) of the UPLC-MS/MS method for the determination of DBP in rat feces.

Nominal concentration (μg/g)	Precision (R.S.D. %)	Accuracy (Bias %)
**Inter-day**		

0.125	4.89	11.93
0.25	11.31	−1.38
0.5	11.86	−3.58
1.25	8.03	−3.71
2.5	1.05	0.11

**Intra-day**		

0.125	17.50	14.23
0.25	9.88	4.17
0.5	7.43	3.48
1.25	4.50	−6.43
2.5	0.82	1.39

**Table 3:** Matrix effect (ME) and recovery (RE) data for DBP and chrysin (IS) in rat plasma and feces.

Nominal concentration	Set 1	Set 2	Set 3	ME (%)	RE (%)
DBP in plasma (μg/mL)					

0.125	18030 ± 469	17659 ± 951	17041 ± 533	97.89	96.58
1.25	149945 ± 4210	147095 ± 3886	148575 ± 11081	98.94	100.92
2.5	285965 ± 2679	282224 ± 1116	280990 ± 6802	98.69	99.57

Average	-	-	-	98.50 ± 0.54	99.02 ± 2.22

IS (1 μg/mL) in Plasma	146906 ± 6353	156220 ± 5833	158258 ± 6815	106.38 ± 2.03	107.73 ± 1.20

DBP in feces (μg/g)					

0.125	17662 ± 554	17791 ± 722	17535 ± 187	100.7	98.63
1.25	154574 ± 3608	152536 ± 1775	152825 ± 2346	100.65	100.18
2.5	309409 ± 4973	311060 ± 2234	309193 ± 5762	100.54	99.39

Average	-	-	-	100.63 ± 0.08	99.4 ± 0.77

IS (1 μg/mL) in feces	113790 ± 15520	113189 ± 3439	105573 ± 1652	100.70 ± 11.05	93.31 ± 1.7

Data expressed as mean ± SD (*n* = 3); ME (%) = (Set 2/Set 1) × 100%; RE (%) = (Set 3/Set 2) × 100%; Set 1: the peak areas obtained in neat solution standards; Set 2: the peak areas for standards spiked after extraction; Set 3: the peak areas for standards spiked before extraction.

**Table 4:** Pharmacokinetic parameters of DBP in rat plasma after DBP (30 mg/kg, i.v.) administration.

Pharmacokinetic parameters	DBP (30 mg/kg, i.v.)
AIC of one-compartment	(−23.57) ± 3.66
AIC of two-compartment	(−37.09) ± 6.30
A (μg/mL)	6.04 ± 1.31
B (μg/mL)	0.18 ± 0.06
α (1/min)	0.15 ± 0.03
β (1/min)	0.02 ± 0.01
AUC (min μg/mL)	57.8 ± 5.93
t_1/2,α_ (min)	5.77 ± 1.14
t_1/2,β_ (min)	217 ± 131
CL(mL/min/kg)	551 ± 64
MRT (min)	123 ± 78

Data are presented as mean ± SEM (*n* = 5). AUC: the area under the concentration-time curve; t_1/2,α_: half-life of the distribution phase; t_1/2,β_: half-life of the elimination phase; A: the concentration intercept for absorption phase; B: the concentration intercept for distribution phase; α: disposition rate constant for absorption phase; β: disposition rate constant for distribution phase; CL: clearance; MRT: mean residence time.

**Table 5:** Mass spectrometric conditions of DBP and chrysin (internal standard, IS).

Parameters	DBP	Chrysin (IS)
Ionization mode	ESI (+)	ESI (+)
Capillary voltage (kV)	3.11	3.11
Cone voltage (V)	22	66
Collision energy (eV)	14	32
Desolvation temperature (°C)	400	400
Desolvation gas flow (L/h)	800	800
Multiple reaction monitoring	*m*/*z* 279.25 > 148.93	*m*/*z* 255.09 > 102.96
